# Supplementary material for: Vaccine Preventable Zoonotic Diseases: Challenges and Opportunities for Public Health Progress
Source: Vaccines (Basel). 2022 Jun 22;10(7):993. doi: 10.3390/vaccines10070993 (PMC9319643; doi:10.3390/vaccines10070993)
Supplement: Supplementary file 1 [file vaccines-10-00993-s001.zip › vaccines-1747768-SI.pdf]

Table S1. Burden of vaccine-preventable zoonoses and vaccine availability.

Pathogen	Impact of Human Disease		Transmission Routes	Human Vaccine Status		Animal Vaccine Status		Animal Species Affected
	Domestic	Global		Domestic	Global	Domestic	Global	
Bacillus anthracis (anthrax)	127 cases were identified annually in the early part of the 20th century, and have subsequently declined to less than 1 case per year—a rate maintained for the past 20 years [100]	Between 20,000 and 100,000 cases identified annually [101]	Direct contact, food-borne, airborne	Licensed for preexposure prophylaxis [102]	Licensed [103]	Licensed	Licensed [104]	Livestock
Bartonella henselae (cat scratch disease)	Estimated 12,500 cases/year [105]	Not available	Direct contact (scratch)	Not available	Not available	Not available	Not available	Feline
Borrelia burgdorferi (Lyme disease)	30,000 cases annually; however, estimates based on insurance records suggest that each year approximately 476,000 Americans are diagnosed and treated [92]	Global burden of disease is not well characterized	Vector-borne (*Ixodes* ticks)	Investigational [106]	Not available	Licensed (canine) Investigational (wildlife)	Licensed (Canada)	Canine Peromyscus mice
Brucella spp. (Brucellosis)	80–120 cases per year [16]	>500,000 cases reported annually [107]	Direct contact with infected material (placenta, aborted fetuses, etc.), food-borne	Not available	Not available	Brucella abortus and B. melitensis Licensed Investigational (wildlife)	Licensed [108]	Bovine, caprine, ovine, wildlife (bison, elk), swine, canine
Burkholderia mallei (glanders)	Zero cases since 1934 [109]	Not available	Direct contact, airborne	Investigational [110]	Investigational [111]	Not available	Not available	*Burkholderia mallei* (glanders)
Campylobacter jejuni	Estimated 845,024 cases per year [111]	7.5 million disability-adjusted life years in the 2010 Global Burden of Disease Study [112]	Food-borne, waterborne, direct contact	Not available	Not available	Vaccines are not available for enteritis but can prevent abortions in sheep; they are	Vaccines are not available for enteritis but can prevent abortions in	Chicken, sheep, cow

						also useful for both prophylaxis and treatment in bovine genital campylobacteriosis; however, vaccinated cows may remain carriers [113]	sheep; they are also useful for both prophylaxis and treatment in bovine genital campylobacteriosis; however, vaccinated cows may remain carriers [113]	
Chlamydophila abortus (chlamydiosis)	Not well characterized	Not available	Direct contact	Not available	Not available	Licensed	Licensed	Caprine, ovine, cats
Chlamydophila psittaci (parrot fever)	Since 2010, there have typically been fewer than 10 cases reported annually [114]	Not available	Direct contact, airborne	Not available	Not available	Not available	Not available	Psittaciform pet birds, poultry
Coxiella burnetti (Q fever)	In 2019, 178 acute and 34 chronic cases were reported [115]	Global burden of disease is not well characterized	Aerosol, direct contact, foodborne	Not available	Licensed (Australia) [116]	Not available	Licensed (European Union) [117]	*Coxiella burnetti* (Q fever)
Cryptosporidia parvum (cryptosporidiosis)	An estimated 322,000,000 cases attributed to water occur annually [118]	Not well characterized	Direct contact, food-borne, waterborne	Not available	Not available	Not available	Not available	Cryptosporidiosis (*Cryptosporidia parvum*)
Eastern equine encephalitis virus	Average of 11 cases/year [119]	Not available	Vector-borne	Investigational [120]	Investigational [121]	Licensed	Licensed [122]	Equine, donkey, mule; off-label use in camelids, emus
Zaire ebolavirus (Ebola virus disease)		Sporadic outbreaks; 28,639 suspected,	Direct contact	Licensed [124]	Licensed [125]	Not available	Not available	Non-human primates and bats [126]

			probable, and confirmed cases of Ebola and 11,316 deaths from Ebola during the 2014 epidemic in West Africa [123]						
Escherichia coli (food-borne)		Estimated 205,781 food-borne E. coli infections annually [111]	Estimated >300 M illnesses annually [127]	Food-borne, water-borne, direct contact, indirect contact, fomite	Investigational [128]	Not available	Licensed	Licensed [129]	Bovine
Hendra virus			Between 1994 and 2013, 7 human cases were reported [130]	Direct contact	Not available	Investigational [131]	Not available	Licensed (Australia) [132]	Equine
Influenza virus	Avian Swine	Avian: four human infections identified in the US since 2002 [133]<br>Swine: from April 12, 2009 to April 10, 2010, CDC estimated there were 60.8 million cases (range: 43.3–89.3 million), 274,304 hospitalizations (range: 195,086–402,719), and 12,469 deaths (range: 8868–18,306) in the United States due to the (H1N1)pdm09 virus [134]	Avian: approximately 45 cases annually [135]<br>Swine: CDC estimated that 151,700–575,400 people worldwide died from (H1N1)pdm09 virus infection during the first year the virus circulated.	Air-borne, direct contact, indirect contact (e.g., contaminated environments)	Licensed [136]	Licensed [137]	Licensed [138]	Licensed [139]	Poultry, swine (ducks, chickens, pigs, horses, dogs, cats, whales, and seals)
Japanese encephalitis virus			An estimated 68,000 cases and 15,000 deaths occur yearly [140]	Vector-borne	Licensed	Licensed	Investigational [141]	Licensed	Equine

Leishmania infantum		An estimated 700,000 to 1 million new cases of Leishmania occur annually [142]	Vector-borne	Investigational [143]	Investigational [144]	Not available	Vaccines available in some countries [145]	Canine
Leptospira spp.	100–200 cases/year [146]	Estimated >1 M cases/year [146]	Contact with water contaminated with the urine of infected animals through cuts/abrasions of the skin or through mucous membranes (eyes, nose, mouth)	Not available	Licensed (China) [147]	Licensed	Licensed [148]	Canine, swine, bovine
MERS-CoV		242 cases/year [149]	Direct or indirect contact—not fully understood	Investigational [150]	Investigational (Germany) [61]	Investigational (in chimps) [151]	Not available	Camel
Mycobacterium bovis	*M. bovis* causes less than 2% of the total number of cases of TB disease [152]	Not available	Food-borne, air-borne	BCG vaccine for infants and young children [153]	BCG vaccine for infants and young children [153]	Investigational [154]	Investigational [154]	Badger, deer, marsupials, wild boar
Nipah virus		Cases occur sporadically [155]	Direct contact, food-borne	Not available	Not available	Investigational [156]	Investigational [156]	Caprine, ovine, bovine
Pasteurella multocida	Not well characterized [157]	Not available	Direct contact, possibly air-borne	Not available	Not available	Licensed [158]	Licensed [158]	Caprine, ovine, bovine, swine, poultry, rabbit
Rabies virus	1–3 deaths per year [159]	59,000 deaths annually [14]	Direct contact (bite,	Licensed [160]	Licensed [6]	Licensed oral	Licensed oral	Wildlife (coyote, gray fox, red fox,

			contamination of mucous membranes such as eyes, nose, mouth), airborne			Licensed parenteral	Licensed parenteral [161]	raccoons, raccoon dogs, skunks), canine Canine, feline, ferret, equine, livestock, camelids
Rickettsia rickettsii (Rocky Mountain spotted fever)	In 2019, 5207 cases of spotted fever group rickettsioses, which includes infections of *R. rickettsii,* were reported in the US [162]	Burden of disease is not well characterized; distributed broadly, albeit reported at a low frequency, throughout the Western Hemisphere [163]	Tick-borne	In development [164]	Not available	Not available, used as a model for human vaccine development	Not available	Primarily dogs
Rift Valley fever virus		Global burden of disease is not well characterized	Direct contact, vector-borne	Not available	Investigational [165]	Not available	Licensed [166]	Livestock (cattle, buffalo, sheep, goats, and camels)
Salmonella (food-borne)	Estimated 1,027,56 non-typhoidal cases annually [111]	Estimated 80.3 M cases annually [167]	Food-borne, direct contact, indirect contact, fomite	Investigational [168]	Licensed [169]	Licensed [170]	Licensed [129]	Poultry, bovine, swine; however, animal vaccines are limited in use
SARS-CoV-2	Over 78 M confirmed cases and 900,000 deaths (as of March 2022) [171]	Over 430 M cases and 5.9 M deaths (as of March 2022) [171]	Aerosol	Licensed: Pfizer and Moderna EUA: Johnson & Johnson	Licensed in various countries: Moderna, Pfizer, Johnson & Johnson, AstraZeneca, Novovax,	Not licensed; experimental use with Zoetis vaccine authorization in mink and zoo animals [67]	Licensed (Russia) [173]	Companion animals, farmed mink, zoo and aquaria animals, wildlife

					Covovax, Covishield, Covaxin, Sinopharm, Sinovac [172]			
Toxoplasma gondii	Estimated that 11% of the population 6 years and older have been infected with Toxoplasma [174]	In various places throughout the world, it has been shown that more than 60% of some populations have been infected with Toxoplasma [174]	Food-borne, water-borne, direct contact, indirect contact (environmental contamination)	Not available	Not available	Not available	Experimental (cats) [175]	Feline, ovine
Venezuelan equine encephalitis virus		Outbreaks have been reported in the Americas since 1935; the largest outbreaks caused approximately 75,000 human cases [176]	Vector-borne	Investigational	Investigational	Licensed	Licensed (in horses)	Equine, donkey, mule
Vesicular stomatitis virus		Not available	Direct contact	Not available	Not available	Not available	Commercial vaccines are available in some endemic regions of Central and South America.	Equine, bovine, swine
Western equine encephalitis virus	Since 1964 there have been fewer than 700 confirmed cases [177]	Not available	Vector-borne	Investigational [178]	Investigational [179]	Licensed	Licensed [122]	

West Nile virus	Over the 20-year existence of WNV in the US (between 1999 and 2019), a total of 51,702 cases were reported to CDC [180]; the total societal costs of WNV hospitalized cases and deaths as reported to CDC for 1999–2012 was estimated to be roughly USD 778 million dollars or ~USD 56 million per year [181]	Global burden of disease is not well characterized	Vector-borne	Investigational [182]	Investigational [77]	Licensed	Licensed [183]	Equine
Yersinia pestis (plague)	Not available	1000–2000 cases are reported to the WHO each year, though the true burden of disease is likely much higher [184]	Direct contact, vector-borne	Investigational [184]	Investigational [185]	Vaccination used to protect endangered black-footed ferrets; vaccines in food bait were given to prairie dogs; no vaccination is approved for domestic species		Prairie dogs, ferrets, rodents
